# Genome-Wide Profiling of Transcriptome and DNA Methylome in Human Embryonic Stem Cells Exposed to Extractable Organic Matter from PM2.5

**DOI:** 10.3390/toxics11100840

**Published:** 2023-10-06

**Authors:** Jianming Wang, Tiantian Liu, Jin Wang, Tao Chen, Yan Jiang

**Affiliations:** 1 Suzhou Medical College, Soochow University, Suzhou 215123, China; 2 Education Key Laboratory of Geriatric Diseases and Immunology, Suzhou 215123, China

**Keywords:** PM2.5, human embryonic stem cells, transcriptome, DNA methylome, toxicity

## Abstract

Increasing evidence indicates that PM2.5 exposure disrupts early embryonic development, but the mechanisms remain unclear. We hypothesized that PM2.5 cause abnormal embryonic development by interfering with DNA methylation and mRNA expression. In this study, we observed that human embryonic stem cells (hESCs) treated with extractable organic matters (EOM) from PM2.5 concentrations above 100 μg/mL exhibited reduced viability. While EOM within non-cytotoxicity concentrations did not affect the expression levels of pluripotency genes, it did enhance cellular proliferation, as indicated by increased Edu incorporation and the upregulation of cell cycle genes (Cdk2, Mdm2). Additionally, EOM significantly influenced the transcriptome patterns in hESCs. Notably, the differentially expressed genes were found to be significantly enriched in processes such as extracellular matrix organization, cell–cell junction organization, chromatin organization, and DNA methylation. Furthermore, we observed whole genomic-wide DNA methylation changes. Through a cross-analysis of changes in DNA methylation and mRNA expression, we identified an enrichment of terms related to the VEGFR signaling pathway and extracellular matrix. The gene signal transduction networks revealed that crucial hubs were implicated in cell growth and division. In conclusion, our findings demonstrate that PM2.5 induce significant alterations in transcriptome and DNA methylome in hESCs, leading to aberrant cell proliferation. This research provides novel insights into the molecular mechanisms underlying the developmental toxicity of PM2.5.

## 1. Introduction

PM2.5 refers to fine particles with an aerodynamic equivalent diameter less than 2.5 micrometers. Compared with coarse particles, PM2.5 have a higher capacity to absorb toxic substances and penetrate deep into the blood circulation, exerting a more severe threat to human health [1]. Moreover, PM2.5 can absorb virous chemicals, among which polycyclic aromatic hydrocarbons (PAHs), known as endocrine disruptors, attract much attention due to their mutagenicity and carcinogenicity [2]. Apart from causing cardiopulmonary diseases and lung cancer, PM2.5 also has adverse effects on embryonic development, resulting in premature birth and stillbirth [3,4,5]. However, the mode of action of the developmental toxicity of PM2.5 remains largely unknown.

Embryos’ early development depends on the precise temporal and spatial regulation of gene expression [6]. Previously, we have demonstrated that extractable organic matters (EOM) from PM2.5 dysregulate the expression patterns of genes involved in oxidative stress, apoptosis and cell differentiation, leading to inhibited cell proliferation and interrupted heart-specific differentiation in mouse teratoma stem cell models and zebrafish embryos [7,8]. The underlying mechanisms of PM2.5-induced gene expression changes warrant further investigation.

DNA methylation is an essential epigenetic mechanism that plays a crucial role during embryonic development [9]. It involves the addition of a methyl group to cytosine residues at CpG dinucleotides, and generally leads to the repression of gene expression. During embryonic development, DNA methylation patterns undergo dynamic changes that are critical for proper cellular differentiation and tissue specialization, and aberrant DNA methylation changes have been linked with birth defects [10,11]. In our previous studies, it has been well documented that DNA methylation is susceptible to environmental pollutes, including PM2.5 [12]. Until now, the PM2.5-induced DNA methylation changes during human early embryonic development have remained unclear.

Currently, the investigation of developmental toxicity induced by PM2.5 is primarily based on animal models such as zebrafish, rats, and mice. However, it is important to consider that species differences may potentially introduce inaccuracies [13,14,15]. It is thus critical to utilize experimental materials derived from humans for translational medicine research. Human embryonic stem cells (hESCs) possess unique characteristics, including unlimited proliferation and the ability to differentiate into all cell types of three germ layers in vitro, which makes them a valuable in vitro model for assessing the developmental toxicity of environmental contaminants [16]. In our previous study, we utilized hESCs as a model to investigate the impact of water pollutants on early embryonic development [17].

We suspected that maternal PM2.5 exposure might dysregulate gene expression via DNA methylation during early embryonic development. Therefore, an in-depth exploration of genome-wide DNA methylation patterns, along with transcriptional analysis, will help to reveal the effects of PM2.5 on developmental toxicity. In the present study, we examined the adverse effects of EOM from PM2.5 on transcriptomic and DNA methylome patterns in hESCs. The correlations between DNA methylation and mRNA expression were investigated and the enriched signaling pathways were identified.

## 2. Materials and Methods

### 2.1. PM2.5 Collection and EOM Extraction

PM2.5 was collected in urban Suzhou, as reported previously [18]. Briefly, samples were collected on quartz films using a Tianhong TH-150C PM2.5 sampler (Wuhan, China). Prior to sampling, the film was heated in a 500-degree electric furnace for 2 h to eliminate volatile components. After weighing, the quartz membrane was placed inside a Soxhlet extraction apparatus along with dichloromethane for an 8 h extraction period. This allowed the PM2.5 particles to dissolve in the organic solvent. Subsequently, a rotary evaporator was employed to remove the majority of the solvent. Once the organic solvent had completely evaporated, an appropriate amount of DMSO was added. After being dissolved in DMSO, the extracts (EOMs) were pooled and stored at −80 °C for further experiment.

### 2.2. Human Embryonic Stem Cell Culture

Human embryonic stem cell line H1 was obtained from Shanghai Cell Bank, Chinese Academy of Sciences. H1 cells were cultured in Matrigel (Corning)-coated 6-well plates in mTeSR1 medium (Stem cells Technologies) at 37 incubator (5% CO_2_).

### 2.3. Cell Viability Assay

Cells were seeded in 96-well plates coated with Matrigel at a density of 2 × 10^4^/cm^2^ supplemented with 10 μM ROCK inhibitor (Y-27632, Millipore). After 24 h, cells were treated with EOM at indicated concentrations for 2 days before being incubated in 0.5 mg/mL (4,5-dimethylthiazol-2-yl) -2,5-diphenyl tetrazolium bromide (MTT, Sigma-Aldrich) at 37 °C for 2 h. The formazan granules generated by live cells were dissolved in dimethyl sulfoxide (DMSO), and the absorbance at 540 nm was monitored by using a multi-well reader.

### 2.4. Immunofluorescence Staining

To detect the protein content of NANOG, OCT4 and SOX2, hESCs were inoculated in a 12-well plate containing a cover glass for 24 h before being treated with EOM at different concentrations (1 μg/mL, 5 μg/mL and 10 μg/mL) for 72 h. Cells were then fixed with 4% paraformaldehyde for 15 min, permeated with 0.2% triton X-100 for 10 min, and blocked with 1% BSA for 1 h. After being probed with primary antibodies (NANOG, Abways Technology; SOX2, Abways Technology; OCT4, Santa Cruz Biotechnology) at 4 °C overnight, the samples were incubated with secondary antibodies (Alexa Fluor 594 and Alexa Fluor 488, Abways Technology) and DAPI for 1 h. Images were taken using a fluorescent inverted microscope (Nikon, Tokyo, Japan).

### 2.5. RNA Preparations and Quantitative Real Time PCR

RNA was extracted and collected by using TRIzol solvent (Tiangen, Beijing, China). Briefly, 1 μg of total RNA was used as a template to synthesize cDNA using RevertAid™ First Strand cDNA Synthesis Kit (Thermo Scientific, Shanghai, China) according to the instructions of the manufacturer. qPCR was performed in an ABI QuantStudio 6 qPCR system (Applied Biosystems, USA) using SYBR Green qPCR Master Mix (Biori, Zhuhai, China). Primers are listed in Table 1. GAPDH was used as a reference gene for the normalization of the expression changes. Relative expression levels of target genes were calculated using the 2^−ΔΔct^ method.

### 2.6. Genomic-Wide mRNA Expression Analysis

Total RNAs from control and EOM-treated samples (1 μg/mL, 5 μg/mL and 10 μg/mL) were isolated. Agilent Human8X60K microarray was used to perform the transcriptomic profiling. The upregulated or downregulated expressed genes with *p* < 0.05 and fold change (FC) > 2 were identified as differentially expressed genes (DEGs). Gene ontology (GO) and pathway analysis were conducted using the DAVID database.

### 2.7. Genomic-Wide DNA Methylation Profiling

Whole genomic DNA was extracted from control or EOM-treated samples (10 μg/mL) to construct the library for MethylRAd sequencing (Hiseq X Ten platform, Illumina, San Diego, CA, USA), as we previously reported [12]. The DNA signatures containing the CCGG and CCWGG sites were extracted from the genome as the reference DNA sequence. A total of 128,725,230 clean reads were generated from hESCs and a total of 1,048,575 methylated CCGG sites were identified. Differential methylation sites (DMSs) and differential methylation genes (DMGs) were screened and identified before GO and KEGG pathway analyses were performed.

### 2.8. Statistical Analyses

All data were presented as the mean of three independent replicates with a standard error (±SEM). Statistical analysis was performed using GraphPad Prism 8.0 software. A t-test was used for the comparison of two groups, and one-way analysis of variance (ANOVA) was used for the comparison of multiple groups. A difference was considered statistically significant with a *p* < 0.05.

## 3. Results

### 3.1. Effects of EOM on Viability and Characteristics of hESCs

**Figure 1:**
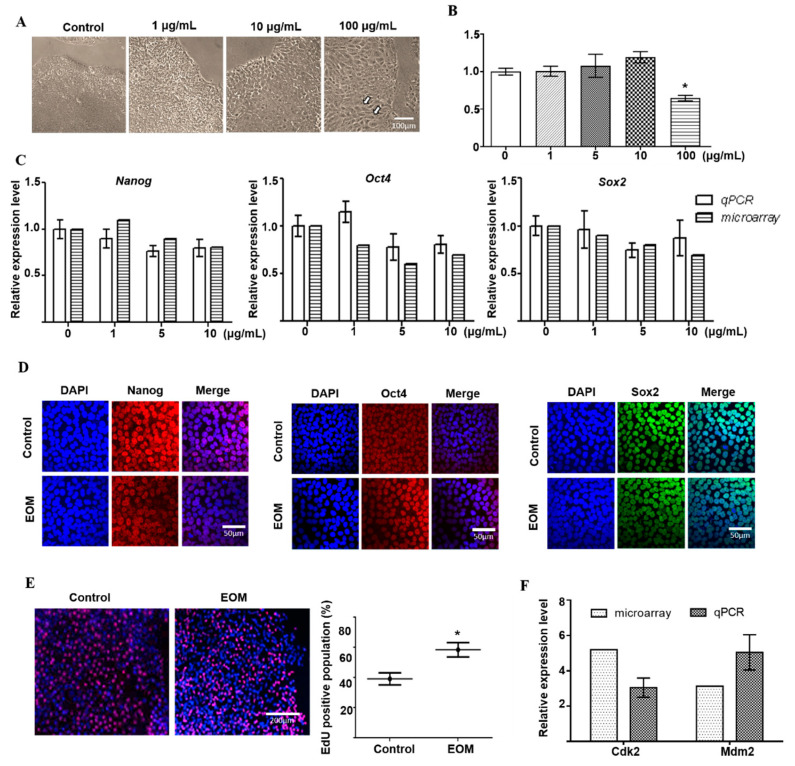
Effects of EOM on hESCs’ morphology, viability, pluripotency and proliferation. (**A**) Representative phase contrast images of human embryonic stem cells in the absence or presence of EOM (with different concentrations: 1 μg/mL, 10 μg/mL, 100 μg/mL); scale bar = 100 μm; (**B**) MTT assay: the effect of different concentrations of EOM on human embryonic stem cells following a 48 h treatment period, with DMSO (0.1%) as negative control and triton X-100 (0.05%) as positive control; (**C**) after 72 h exposure to EOM (with different concentrations: 1 μg/mL, 5 μg/mL, 10 μg/mL), cells were subjected to microarray analysis for the mRNA expression of genes related to pluripotency, followed by q-PCR verification; (**D**) cells were exposed to EOM (10 μg/mL) for 72 h and subjected to immunofluorescence staining for Nanog (red), Oct4 (red) and Sox2 (green), with nuclear counterstaining with DAPI (blue), scale bar: 50μm; graph represents the relative fluorescence intensity quantification; (**E**) EdU incorporation assay was used to assess the DNA synthesis, and DAPI staining (blue) represents cell nuclei. EdU-stating (red) spots represent cells undergoing DNA synthesis. Graph represents the percentage of EdU-positive cells based on total DAPI-stained cells and indicates mean values ± SE, scale bar:200 μm; (**F**) the expressions of Cdk2 and Mdm2 were measured using microarray and q-PCR. Samples were collected from at least three independent experiments, * *p* < 0.05.

After 72 h EOM exposure, noticeable changes in cell morphology were observed, as depicted in Figure 1A. EOM-treated cells showed less compacted colonies and increased intercellular space in a dose dependent manner, as indicated by the white arrow (Figure 1A). Additionally, individual cells underwent a remarkable transformation from small and round to large and polygonal shapes, accompanied by visible nucleoli (Figure 1A). To assess the impact of EOM concentration on cell viability, we performed an MTT test using EOM with varying concentrations. As shown in Figure 1B, cells treated with EOM at 100 μg/mL exhibited a considerable decrease in viability, indicating the presence of cytotoxicity induced by EOM concentrations above 100 μg/mL. Hence, for subsequent experiments, EOM concentrations below 100 μg/mL were used to exclude cytotoxic effects. Microarray analysis followed by q-PCR verification demonstrated that the expression of pluripotency-associated genes, including Nanog, Oct4, and Sox2 (Figure 1C), did not show significant alterations upon EOM treatment (Figure 1C). EOM did not affect the distribution or intensity of pluripotency markers, evidenced by immunofluorescence staining (Figure 1D). However, EOM exerted a significant impact on cell proliferation. The percentage of EdU-positive cells was 39.2 ± 4.0% in the control group, and 58.5 ± 4.7% in EOM group (*p* < 0.05, Figure 1E). Further analysis, as depicted in Figure 1F, revealed that EOM regulated cell proliferation partly through upregulating CDK2 and MDM2, which was validated by both microarray data and q-PCR verification.

### 3.2. EOM-Induced Transcriptomic Changes in hESCs

**Figure 2:**
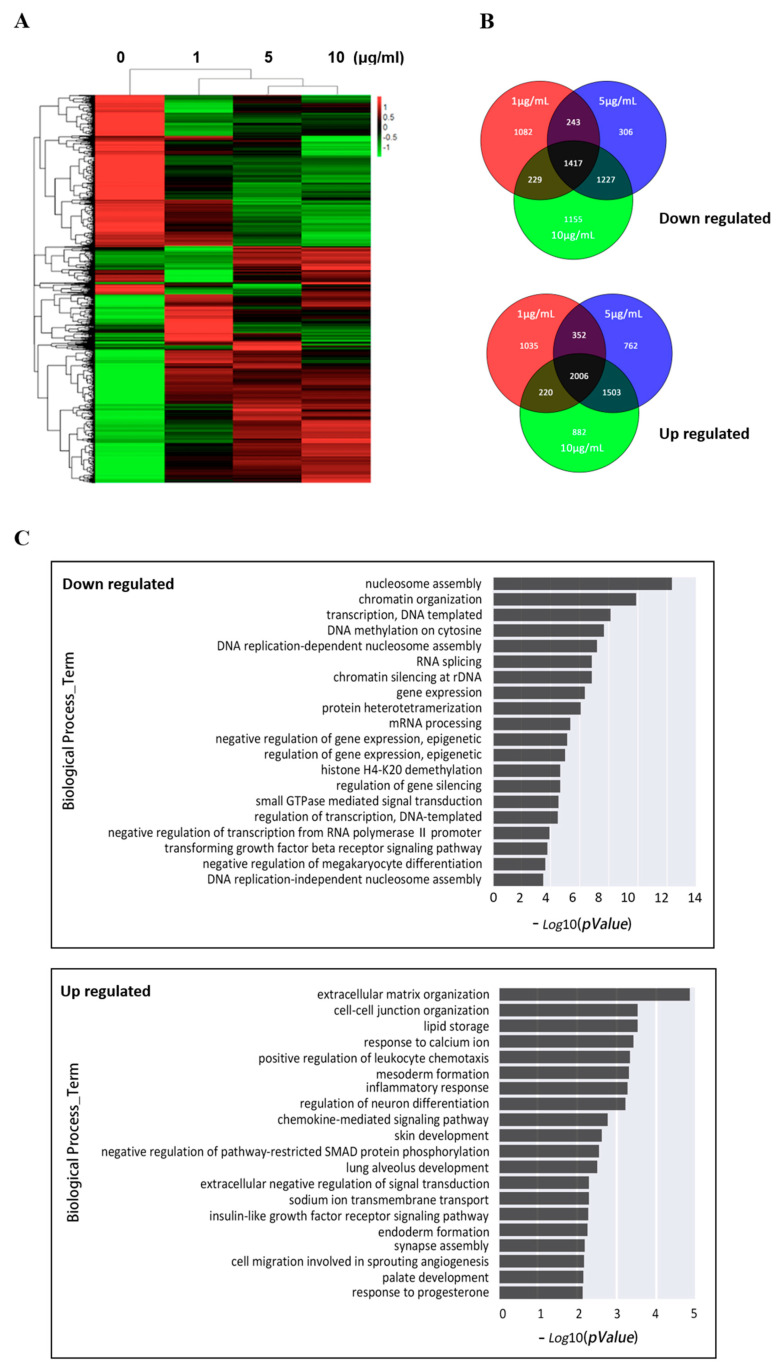
Effects of EOM at different concentrations on transcriptome in hESCs. (**A**) Heatmap displaying the expression levels of genes that were differentially expressed following treatment with various concentrations (1 μg/mL, 5 μg/mL, 10 μg/mL) of EOM; (**B**) Venn diagram of differentially expressed genes (DEGs) resulting from EOM treatment—upper: downregulated, lower: upregulated; (**C**) KEGG analysis was performed to enrich the pathways associated with down-regulated and up-regulated differentially expressed genes.

To identify genes affected by EOM, we performed a genome-wide transcriptional analysis. Even at the lowest dose level of 1 μg/mL, EOM induced extensive transcriptional changes compared with the controlled group (Figure 2A). The further analysis of transcriptional levels revealed that 3613 genes were up-regulated and 2971 genes were down-regulated in the group treated with 1μg/mL EOM. In the group treated with 5 μg/mL EOM, 4623 genes were up-regulated and 3193 genes were down-regulated, while in the group treated with 10 μg/mL EOM, 4611 genes were up-regulated and 4028 genes were down-regulated. When intersecting the microarray data from different concentrations of EOM (1, 5, 10 μg/mL), our results demonstrated that EOM exposure caused 2006 genes to be upregulated while 1417 genes were downregulated, all exhibiting fold change > 2, as demonstrated in Figure 2B. These DEGs were further investigated for their function using Gene Ontology analysis. As shown in Figure 2C, the downregulation DEGs were significantly enriched in the biological process related to extracellular matrix organization, cell–cell junction organization, which aligns with the observed abnormal phenotypic changes such as cell junction alterations (Figure 1A). On the other hand, the upregulated DEGs were prominently associated with the DNA replication process. Interestingly, the enrichment analysis also revealed an association with chromatin organization and DNA methylation (Figure 2C).

### 3.3. EOM Changed the DNA Methylation Patterns in hESCs

**Figure 3:**
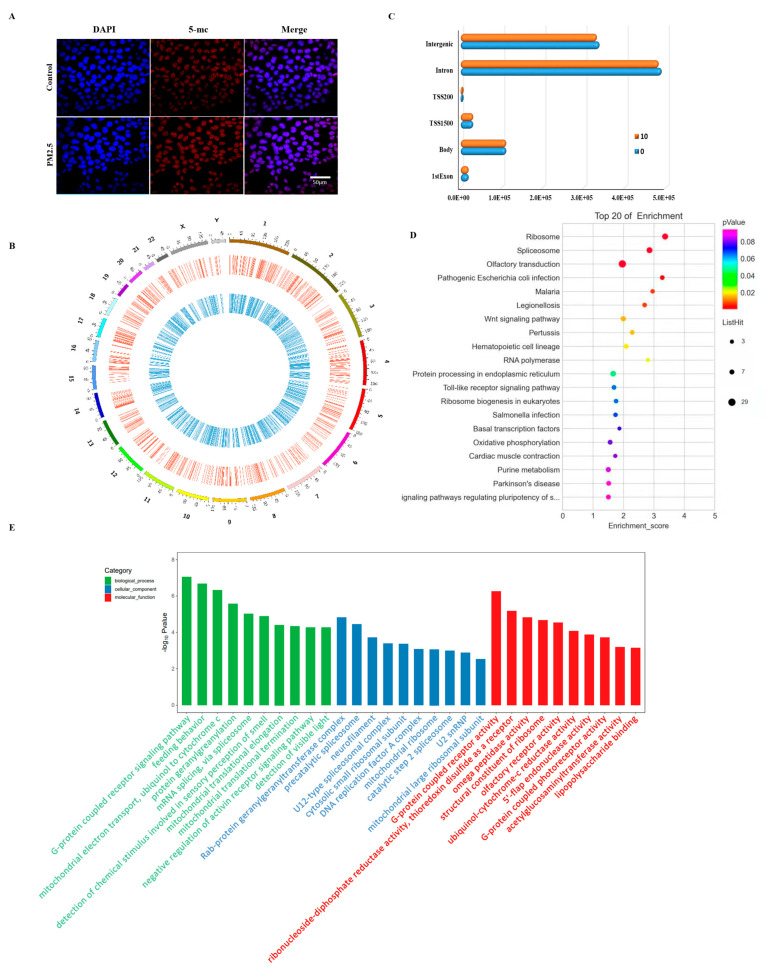
Effect of EOM exposure on DNA methylome patterns. (**A**) Representative images of immunofluorescence staining against 5-mC antibody in the absence or presence of EOM (10 μg/mL), EOM exposure, blue: DAPI; red: 5-mC, scale: 50 μm; (**B**) distribution of differentially methylated sites on different chromosomes—red: hypermethylated sites, blue: hypomethylated sites; (**C**) distribution of differential methylation sites on different functional components; (**D**) KEGG enrich analysis of differentially methylated genes involved in different signaling pathways; (**E**) GO enrichment analysis of differentially methylated genes, including cellular component, molecular function and biological process.

Since transcriptomics data indicated that DNA methylation might be affected by EOM treatment, we conducted immunofluorescence staining against the 5-mC antibody to assess the global genome DNA methylation changes. As demonstrated in Figure 3A, there was a significant increase in total 5-mC levels in EOM samples compared to untreated control cells. To gain further insights, we employed a Methyl-RAD assay to examine whole genomic-wide DNA methylation changes. The average sequencing depths of these methylation sites were 27.59% in the control group and 29.99% in the treated group. As shown in Figure 3B, EOM at 10 μg/mL induced substantial and widespread changes in DNA methylation. The majority of differentially methylated sites were located in intergenic and intron regions, accounting for 34.67% and 54.53%, respectively (Figure 3C), while only a relatively smaller portion was detected in promoter regions. To explore the functional implications of these DNA methylation changes, GO and KEGG pathway enrichments were performed. Genes associated with differentially DNA-methylated sites were enriched in MAPK signaling pathways (Figure 3D,E).

### 3.4. Integrated Analysis of Differentially Methylated Genes and Differentially Expressed Genes after EOM Exposure

**Figure 4:**
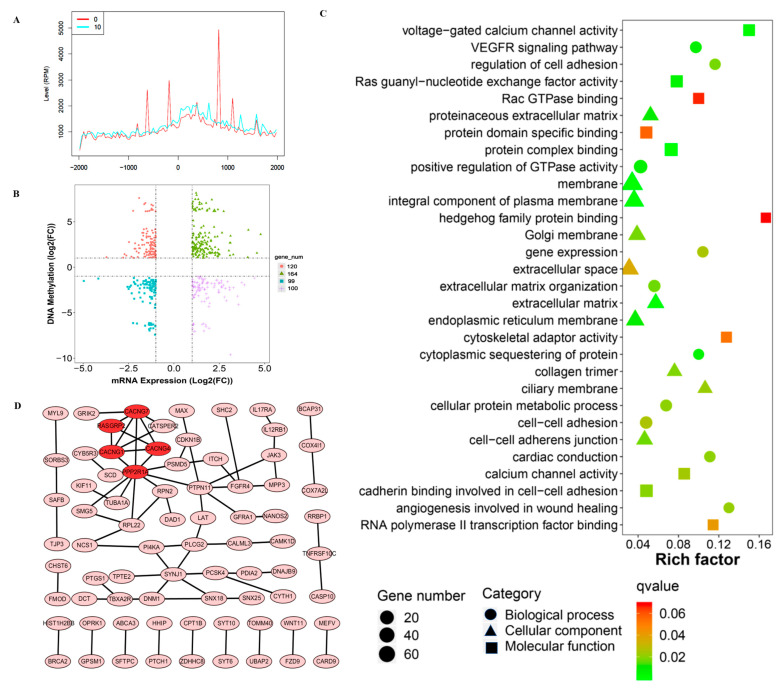
Cross-analysis of differentially expressed genes, differentially methylated genes and PPI Network. (**A**) The location of RPM peaks; (**B**) correlation analysis of differentially expressed genes and differentially methylated genes; (**C**) GO analysis of overlapping genes with negative correlations; (**D**) protein–protein and protein–DNA interaction analysis.

As alterations in DNA methylation levels near promoter regions have been commonly correlated with gene expression levels, we conducted a cross-analysis of DNA TSS 2000 in DMGs and mRNA expression level in DEGs. As shown in Figure 4A, the RPM peaks located a coverage depth of upper and lower 1000 bp around the transcriptional start sites. A total of 483 overlapping genes were identified between DEGs and DMGs, as illustrated in Figure 4B. Among these genes, a subset of 220 exhibited negative association. Specifically, 120 genes displayed a hypermethylation of DNA accompanied by the downregulation of mRNA, while 100 genes showed a hypomethylation of DNA along with the up-regulation of mRNA. Conversely, 263 genes demonstrated a positive association, whereby hypermethylation was linked with up-regulation in 164 genes, and hypomethylation was linked with down-regulation in 99 genes. We then further categorized the overlapping genes with negative association. GO analysis revealed the top 10 terms in enrichment related to biological process, cellular component and molecular function, as shown in Figure 4C. Notably, the enriched terms encompassed various processes such as voltage-gated calcium channel activity, VEGFR signaling pathway and extracellular matrix, etc. (Table 2). In order to explore the protein–protein and protein–DNA interactions, we constructed gene signal transduction networks from those overlapping genes. A total of 76 interaction pairs were identified (Figure 4D). Among them, several hub genes including Calcium Voltage-Gated Channel, PPP2R1A and CDKN1B were identified.

## 4. Discussion

PM2.5 are a common air pollutant known for their toxicity, which is mainly determined by the substances they absorb on their surface. These substances include heavy metals, water-soluble ions and organic compounds. Among these, the organic components are of particular concern due to their high toxicity and relatively consistent levels throughout different seasons and regions [2]. Although the adverse effects of PM2.5 on lung cancer and cardiovascular diseases have been well established, their risk to human embryo development remains a topic of debate. Specifically, organic matter adsorbed onto PM2.5 has garnered attention due to its carcinogenicity and developmental toxicity [19]. Thus, in this study, we aimed to investigate the effects of extractable organic matter from PM2.5 on hESCs, focusing on genome-wide changes in DNA methylation and mRNA expression changes.

In the current study, our findings revealed that even at the lowest concentration tested (1 μg/mL), EOM affected the morphology of hESCs. But EOM at concentrations below 10 μg/mL had minimal impact on cell survival rate, which is consistent with previous reports by Jin and our group [7,20]. However, contrary to Jin’s research, which reported increased reactive oxygen species production and apoptosis, we observed enhanced proliferation in hESCs upon EOM exposure. Previous studies have underscored the dual role of reactive oxygen species, wherein high levels tend to exhibit toxic effects, while moderate levels promote signal transduction and proliferation [21]. Additionally, it is worth noting that Jin utilized whole particles collected from a tire manufacturing plant in their study, whereas in our current investigation we employed an organic extract of PM2.5 collected from an urban area. The differences in ROS levels observed between these two studies may be attributed to variations in PM2.5 dosage, components, exposure duration, or specific cell lines employed.

The precise spatiotemporal regulation of gene expression is crucial for maintaining the characteristics and functions of embryonic stem cells [22]. To the best of our knowledge, there have been no reports on the effects of PM2.5 on the transcriptome of hESCs. Here, we present comprehensive changes in the whole cell transcriptome of hESC following EOM exposure. The differentially expressed genes were significantly enriched in biological processes related to the extracellular matrix and DNA replication. These findings are in line with previous research, indicating that cigarette smoke, which also consists of PAHs, affected adhesion to the extracellular matrix and the proliferation capacity of pre-implantation embryos [23]. Furthermore, our current data demonstrated an upregulation of cell-cycle-related genes (CDK2 and MDM), contributing to the observed increase in cell proliferation induced by EOM [17,24].

Our transcriptomic data revealed a significant upregulation in the expression of genes associated with chromatin organization and DNA methylation following exposure to EOM. Previous studies have extensively documented the ability of environmental pollutants, including PM2.5, to disrupt DNA methylation patterns, leading to the development of various diseases [25,26,27]. In a previous study, we observed substantial changes in DNA methylation in the hearts of zebrafish embryos exposed to EOM [12]. Similarly, in the present study, we observed elevated global DNA methylation levels and modifications in genomic-wide DNA methylation patterns in response to EOM exposure. Through an integrated analysis of DNA methylation and mRNA expression profiles, we identified notable alterations in crucial pathways such as voltage-gated calcium channel activity, VEGFR signaling pathway, and extracellular matrix in EOM-exposed samples. These pathways are essential to early embryonic development [28,29,30]. The protein–protein interaction analysis further highlighted several hub genes involved in cell proliferation regulation, including Calcium Voltage-Gated Channel, PPP2R1A and CDKN1B. Future studies should delve into the functional consequences of the identified gene expression and DNA methylation changes to unravel the underlying mechanisms linking PM2.5 exposure to adverse health outcomes.

## 5. Conclusions

Our data provide compelling evidence that exposure to PM2.5 induces significant alterations in DNA methylation, potentially affecting the expression of key genes crucial for early development. These findings significantly contribute to our understanding of the biological effects of PM2.5 and offer valuable insights for future research in the field of environmental health.

## Figures and Tables

**Table 1:** Primer sequences used.

Genes	Forward (5′-3′)	Reverse (5′-3′)
GAPDH	CAGGAGGCATTGCTGATGAT	GAAGGCTGGGGCTCATTT
OCT4	GGGAGATTGATAACTGGTGTGTT	GTGTATATCCCAGGGTGATCCTC
Nanog	TTTGTGGGCCTGAAGAAAACT	AGGGCTGTCCTGAATAAGCAG
SOX2	TACAGCATGTCCTACTCGCAG	GAGGAAGAGGTAACCACAGGG

**Table 2:** KEGG analysis of DEGs and DMGs with negative correlation.

KEGG Term	Count	%	*p*-Value	Genes
hsa05200:Pathways in cancer	13	6.565657	0.010758	CDKN1B, MAX, HHIP, PTCH1, FZD9, CALML3, BRCA2, RASGRP2, WNT11, PLCG2, IL12RB1, FGFR4, JAK3
hsa04260:Cardiac muscle contraction	5	2.525253	0.013998	CACNG7, COX7A2L, COX4I1, CACNG1, CACNG4
hsa04921:Oxytocin signaling pathway	6	3.030303	0.024599	CACNG7, CAMK1D, CALML3, CACNG1, MYL9, CACNG4
hsa04625:C-type lectin receptor signaling pathway	5	2.525253	0.025232	CLEC4M, CARD9, PLCG2, PTPN11, CALML3
hsa05217:Basal cell carcinoma	4	2.020202	0.029833	WNT11, PTCH1, HHIP, FZD9
hsa04014:Ras signaling pathway	7	3.535354	0.04077	SHC2, PLCG2, PTPN11, CALML3, FGFR4, RASGRP2, LAT
hsa00562:Inositol phosphate metabolism	4	2.020202	0.043319	MIOX, SYNJ1, PI4KA, PLCG2
hsa05214:Glioma	4	2.020202	0.046326	SHC2, CAMK1D, PLCG2, CALML3
hsa04072:Phospholipase D signaling pathway	5	2.525253	0.074274	SHC2, PLCG2, PTPN11, CYTH1, DNM1
hsa04261:Adrenergic signaling in cardiomyocytes	5	2.525253	0.07719	CACNG7, PPP2R1A, CALML3, CACNG1, CACNG4
hsa04934:Cushing syndrome	5	2.525253	0.084729	CDKN1B, WNT11, FZD9, PDE8A, CYP17A1
hsa03015:mRNA surveillance pathway	4	2.020202	0.085772	PPP2R1A, PABPC3, TARDBP, SMG5
hsa04070:Phosphatidylinositol signaling system	4	2.020202	0.085772	SYNJ1, PI4KA, PLCG2, CALML3
hsa04916:Melanogenesis	4	2.020202	0.094103	WNT11, DCT, FZD9, CALML3
hsa04010:MAPK signaling pathway	7	3.535354	0.09527	CACNG7, MAX, SRF, CACNG1, FGFR4, RASGRP2, CACNG4

## Data Availability

Data are available upon request.
